# Macrophage migration inhibitory factor exacerbates asthmatic airway remodeling via dynamin-related protein 1-mediated autophagy activation

**DOI:** 10.1186/s12931-023-02526-y

**Published:** 2023-09-06

**Authors:** Jin Liu, Yuqian Chen, Huan Chen, Yan Wang, Danyang Li, Qianqian Zhang, Limin Chai, Yuanjie Qiu, Jia Zhang, Nirui Shen, Qingting Wang, Jian Wang, Manxiang Li

**Affiliations:** Department of Respiratory and Critical Care Medicine, The First Affiliated Hospital of Xi’an Jiaotong University, No. 277, West Yanta Road, Xi’an, Shaanxi 710061 People’s Republic of China

**Keywords:** Asthma, Macrophage migration inhibitory factor, GTPase dynamin-related protein 1, Autophagy, Airway remodeling

## Abstract

**Background:**

Macrophage migration inhibitory factor (MIF) and GTPase dynamin-related protein 1 (Drp1)-dependent aberrant mitochondrial fission are closely linked to the pathogenesis of asthma. However, it is unclear whether Drp1-mediated mitochondrial fission and its downstream targets mediate MIF-induced proliferation of airway smooth muscle cells (ASMCs) in vitro and airway remodeling in chronic asthma models. The present study aims to clarify these issues.

**Methods:**

In this study, primary cultured ASMCs and ovalbumin (OVA)-induced asthmatic rats were applied. Cell proliferation was detected by CCK-8 and EdU assays. Western blotting was used to detect extracellular signal-regulated kinase (ERK) 1/2, Drp1, autophagy-related markers and E-cadherin protein phosphorylation and expression. Inflammatory cytokines production, airway reactivity test, histological staining and immunohistochemical staining were conducted to evaluate the development of asthma. Transmission electron microscopy was used to observe the mitochondrial ultrastructure.

**Results:**

In primary cultured ASMCs, MIF increased the phosphorylation level of Drp1 at the Ser616 site through activation of the ERK1/2 signaling pathway, which further activated autophagy and reduced E-cadherin expression, ultimately leading to ASMCs proliferation. In OVA-induced asthmatic rats, MIF inhibitor 4-iodo-6-phenylpyrimidine (4-IPP) treatment, suppression of mitochondrial fission by Mdivi-1 or inhibiting autophagy with chloroquine phosphate (CQ) all attenuated the development of airway remodeling.

**Conclusions:**

The present study provides novel insights that MIF promotes airway remodeling in asthma by activating autophagy and degradation of E-cadherin via ERK/Drp1 signaling pathway, suggesting that targeting MIF/ERK/Drp1 might have potential therapeutic value for the prevention and treatment of asthma.

**Supplementary Information:**

The online version contains supplementary material available at https://doi.org/10.1186/s12931-023-02526-y.

## Background

Asthma is a heterogeneous disorder characterized by airway hyper-responsiveness (AHR) and airway inflammation that arise from distinct pathobiological mechanisms [1, 2]. The excessive proliferation, migration, and contraction of airway smooth muscle cells (ASMCs) are the main pathological changes associated with airway wall thickening, airflow obstruction, airway basal resistance and ultimately AHR [3]. Hence, understanding the mechanism of ASMCs proliferation is essential for the prevention and treatment of asthma.

Macrophage migration inhibitory factor (MIF) is a pleiotropic cytokine involved in many autoimmune diseases and chronic inflammatory disorders as a modulator of responses of immune populations and a prominent function in cell survival signaling beyond its proinflammatory function [4, 5]. MIF has been identified as a biomarker of airway remodeling pathogenesis. Li et al. have reported that the serum level of MIF in asthmatic patients significantly increases compared with the healthy individuals [6]. Meanwhile, in ovalbumin (OVA) or house dust mite (HDM) induced asthma rodent models, MIF levels are elevated in circulation, alveolar lavage fluid and lung tissues, and these elevations are associated with enhanced airway remodeling [7, 8]. In addition, MIF promotes proliferation and migration of ASMCs in vitro [6]. Collectively, these studies suggest that MIF plays a crucial role in the pathophysiology of asthma. As a new MIF-specific suicide substrate, 4-iodo-6-phenylpyrimidine (4-IPP), which covalently and irreversibly binds to MIF and inhibits its biological activity [9, 10], has been shown to reduce cell proliferation, migration, invasion and secretion of pro-inflammatory mediators in a variety of diseases [11]. However, the effectiveness of 4-IPP in the treatment of asthma has not been evaluated, which was one of the aims of this study.

Overactivation and upregulation of the GTPase dynamin-related protein 1 (Drp1) have been reported to mediate aberrant mitochondrial fission during asthma development, which further promotes the proliferation of ASMCs [12–14]. Phosphorylation of Drp1 Ser616 has been shown to enhance the GTPase activity of this protein, such activation facilitating Drp1 translocation from the cytoplasm to the mitochondria and interacting with binding partners, thereby promoting mitochondrial fission [15–17]. Many reports have demonstrated that phosphorylation of Drp1 (Ser616) by extracellular signal-regulated kinase (ERK) 1/2 can trigger abnormal mitochondrial fission and promote cell proliferation and migration in a variety of cancers and benign diseases [18–20]. However, to date, whether Drp1 activation mediates MIF-induced ASMCs proliferation, or whether pharmacological inhibition of MIF alleviates mitochondrial dynamic changes involved in airway pathologic changes remains to be unclear.

Autophagy is a highly conserved catabolic process. Feng et al. find that Drp1-mediated mitochondrial fragmentation induces pulmonary remodeling through the activation of autophagy in rat models of monocrotaline-induced pulmonary hypertension [18]. In animal models of asthma and in vitro cultured ASMCs, MIF can increase autophagic activity through activation of Beclin1, leading to proliferation of ASMCs [6], however, the exact molecular mechanism is not yet fully understood. E-cadherin is involved in the structure and immune function of the airway epithelium by regulating epithelial junctions, proliferation and the production of growth factors and pro-inflammatory mediators that modulate the immune response [21]. Recent studies have found that downregulation of E-cadherin expression promotes the proliferation of human aortic smooth muscle cells and a variety of malignant cell lines [22]. Zhai et al. demonstrate that E-cadherin can be degraded by activated autophagy, leading to cell proliferation [23]. Further studies have shown that MIF can downregulate E-cadherin in a variety of cancer cells [24, 25]. Taken together, we assume that extracellular MIF is a key trigger of airway remodeling, which might be mediated by Drp1 Ser616 phosphorylation via the ERK1/2 signaling pathway and subsequently promotes autophagy activation and E-cadherin degradation.

## Materials and methods

### Cell culture and reagents

Primary ASMCs were extracted from the tracheas and main bronchi of naïve male Sprague–Dawley (SD) rats (110–150 g) that were not exposed to ovalbumin (or any other factor) as the method described previously [26]. The isolated smooth muscle layer of the tracheas was cultured with high glucose Dulbecco's modified Eagle medium (DMEM) (Gibco, USA) containing 10% fetal bovine serum (FBS) (Vivacell, China) and penicillin–streptomycin (Genview, USA). Cells were incubated in a humidified incubator with 5% CO_2_ at 37 °C and passaged using 0.25% trypsin (Beyotime, China). ASMCs were used for further experiments between passages 3 and 6. α-smooth muscle actin (α-SMA) (Proteintech, USA, 14395-1-AP, 1:200 dilution) immunofluorescence staining confirmed that the cultured cells contained over 95% ASMCs. ASMCs were serum-starved (1% FBS-DMEM) overnight before each experiment. MIF (Novus Biologicals, USA, NBP2-35276) was used to stimulate ASMCs. U0126 (10 μM) (MedChemExpress, USA) was applied to inhibit ERK1/2, and Chloroquine phosphate (CQ, 20 μM) (Aladdin, China) was employed to inhibit autophagy. The concentrations of the compounds were chosen based on previous studies [6, 18, 27].

### Cell proliferation measurements

Cell viability was measured by CCK-8 kit (GlpBio, USA). Cells were seeded at 2 × 10^3^ per well into 96-well plates for 24 h and then starved with 1% serum overnight followed by incubation with MIF. The cells were incubated with CCK-8 solution (1:10) for 3 h, and then the absorbance was measured at 450 nm using a microplate reader (Bio‐Rad, USA). The incorporation rate of EdU (5-ethynyl-2' -deoxyuridine) was assayed using the BeyoClick™ EdU-488 Kit (Beyotime, China) according to the manufacturer's instructions. Briefly, EDU was added to the culture medium at a final concentration of 10 μM for 2 h, followed by 15 min of fixation and 10 min of permeabilization at room temperature. After incubation in 0.5 ml Click reaction solution for 30 min protected from light, nuclear staining was performed using Hoechst 33,342 for 10 min. Then the images were performed using an inverted fluorescence microscope and the number of EdU-positive cells/total cells were counted using Image J software (NIH, USA).

### siRNA transfection

When ASMCs reached 40%-50% density, transfection was carried out using siRNA dissolved in Lipofectamine ™ 3000 regent (Invitrogen, USA) for 6–8 h, followed by continued incubation in the original medium for 48 h for protein knockdown, and the efficiency of siRNA transfection was detected by Western blotting. All siRNA was synthesized by GenePharma (China). The sequences of siRNA duplexes are as follows: Drp1 siRNA, sense 5′-GGUGCUAGGAUUUAUATT-3′, antisense 5′-UAUAACAAAUCCUAGCACCTT-3′; negative control (NC) siRNA, sense 5′- UUCUCCGAACGUGUCACGUTT-3′, antisense 5′-ACGUGACGUUCGGAGAAT-3′.

### Western blotting

Proteins were isolated by using RIPA lysis buffer (SolarBio, China) for 10 min followed by centrifugation at 12,000 rpm at 4 °C for 15 min. The supernatant was collected as protein samples and separated on 8–12% SDS-PAGE gel and transferred onto PVDF membranes (Millipore, USA). Membranes were probed with the following antibodies against: p‐ERK1/2 (1:2000 dilution, Cell Signaling Technology, USA), t‐ERK1/2 (1:1000 dilution, Cell Signaling Technology, USA), p‐Drp1-Ser616 (1:1000 dilution, Biorbyt, UK), t‐Drp1(1:1000 dilution, Abcam, UK), LC3B (1:1000 dilution, Proteintech, China), P62 (1:1000 dilution, Cell Signaling Technology, USA), ATG5 (1:500 dilution, Proteintech, China), E-cadherin (1:5000 dilution, Proteintech, China) and GAPDH (1:4000, Proteintech, China) at 4 °C overnight, and then re‐blotted with horseradish peroxidase‐labelled secondary antibodies (anti‐mouse, 1:8000, dilution ZhuangzhiBio, China; anti‐rabbit, 1:8000 dilution ZhuangzhiBio, China) at room temperature for 1 h. Bioluminescence was detected by Amersham Imager 600 (GE Healthcare, USA) and quantified by Image J software.

### Animal grouping, modelling and drug administration

All procedures were approved by the Institutional Animal Ethics Committee of Xi'an Jiaotong University and followed the Guide for the Care and Use of Laboratory Animals of the Animal Experimentation Center of Xi'an Jiaotong University. Male SD rats were purchased from the Experimental Animal Center of Xi'an Jiaotong University and housed in an SPF (specific pathogen-free) and controlled temperature (22 ± 2 °C) environment with a 12-h light/dark cycle. The induction of chronic ovalbumin (OVA)-induced asthma model was divided into two stages: sensitization stage: rats (weighing approximately 200 ± 20 g) were injected with 10% OVA solution (1 ml containing 100 mg OVA powder and 100 mg aluminum hydroxide dry powder) at four subcutaneous points (both sides of the abdomen and bilateral groin) and one-point intraperitoneal injection with a total volume of 1 ml on days 0, 7 and 14; nebulization excitation stage: from days 21 to 74, rats were placed in an airtight box with 1% OVA solution for 30 min each time three times a week on alternate days for 8 weeks. In addition, the intervention was started on day 21 of the experiment and was administered 30–60 min before each OVA nebulization excitation for a total of 8 weeks (Fig. 5a). Control rats (n = 5) were administrated with normal saline instead of OVA in both the sensitization and excitation stages. All OVA-sensitized rats were randomly divided into 5 groups (n = 5 rats/group) and treated as follows: OVA model group; OVA + DMSO group: received vehicle DMSO by daily ip injection; OVA + MIF inhibitor 4-IPP group: received 4-IPP (5 mg/kg, Yuan Ye Bio-Technology, China) by ip injection three times a week [28]; OVA + Mitochondrial division inhibitor Mdivi‐1 group: received Mdivi‐1 (50 mg/kg, MedChemExpress, USA) by twice weekly ip injection [29]; OVA + autophagy inhibitor CQ group: received CQ (60 mg/kg, Aladdin, Shanghai, China) by daily gavage tube [30].

### Assessment of airway responsiveness

After 24 h of the last OVA challenge, rats were anesthetized and inserted with a tracheostomy tube. Rats were ventilated using a FlexiVent small animal ventilator (SCIREQ, Canada). After the basal assessment, rats were inhaled with increasing doses of nebulized methacholine chloride (Merck, Germany) (0, 3.125, 6.25, 12.5, 25, 50 and 100 mg/ml) and PBS was used as dilution solvent. To ensure that respiratory strength returned to baseline, there was a 3-min interval between each test. Respiratory resistance (Rrs) was measured using the forced oscillation method to indicate the change in AHR.

### Enzyme-linked immunosorbent assay (ELISA)

The lung tissue homogenate was rinsed with pre-cooled PBS to remove residual blood and then fully ground, and the homogenate was centrifuged at 2–8 °C for 5–10 min at 5000 × g, and the supernatant was taken for testing. The levels of MIF, IL-5 and IL-13 of lung homogenates were determined by ELISA with commercial kits (Elabscience, China), in accordance with instructions of the manufacturer. All results were measured by an absorbance microplate reader at 450 nm.

### Lung histological and immunohistochemistry (IHC) staining

Lung tissues from the right upper lobe margin were fixed overnight at room temperature in 4% paraformaldehyde and embedded in paraffin. Tissue section (5 μm) were stained with hematoxylin and eosin (H&E), periodic acid-Schiff (PAS) and Masson trichrome stains, and all slides were evaluated with light microscopy at a magnification of × 400. H&E staining was used to observe histopathological changes in the lungs. Total bronchial wall area (WAt) and bronchial basement membrane perimeter (Pbm) were measured with Image-Pro Plus software (Media Cybernetics, USA), and bronchial wall thickness (WAt/Pbm) was calculated. Goblet cell proliferation was examined by PAS staining. The area of PAS staining was measured with Image-Pro Plus software. The PAS staining area/airway epithelial area was quantified. Peribronchial collagen deposition was examined by Masson trichrome staining. Airway collagen fiber area (Wcol) and Pbm were measured with Image-Pro Plus software, and the degree of subepithelial collagen fiber deposition (Wcol/Pbm) was calculated [31]. Immunohistochemistry (IHC) staining for α‐SMA (1:200 dilution, Boster, USA) was also performed to detect the degree of bronchial muscularization, as previously described [32]. α-SMA positive area of tracheal wall/Pbm represents the degree of tracheal wall α-SMA expression measured with Image-Pro Plus software. Lung pathology observations and measurements were performed by two independent investigators in a double-blind manner.

### Transmission electron microscopy

Lung tissues from rats were fixed in glutaraldehyde, post-fixed with OsO4, dehydrated in alcohol and then embedded in Aladdin's stone as described previously [33]. Sections of 70 nm were cut from the specimens and stained with uranyl acetate and lead citrate. The mitochondrial morphological structure was assessed using a transmission electron microscope (TEM) (H-7650, Japan).

### Statistical analysis

Data were expressed as mean ± standard error (SEM). All data passed the Shapiro–Wilk test and the F-test for normality and equal variance, respectively. Independent samples t-test was used to compare between the two groups. Comparisons between multiple groups were made using one-way ANOVA followed by Tukey’s multiple comparisons post-hoc test. All statistical analyses were processed using Prism version 8.0 (GraphPad Software, USA). P-values < 0.05 were determined to be statistically significant.

## Results

### MIF induces proliferation of ASMCs

**Fig.1:**
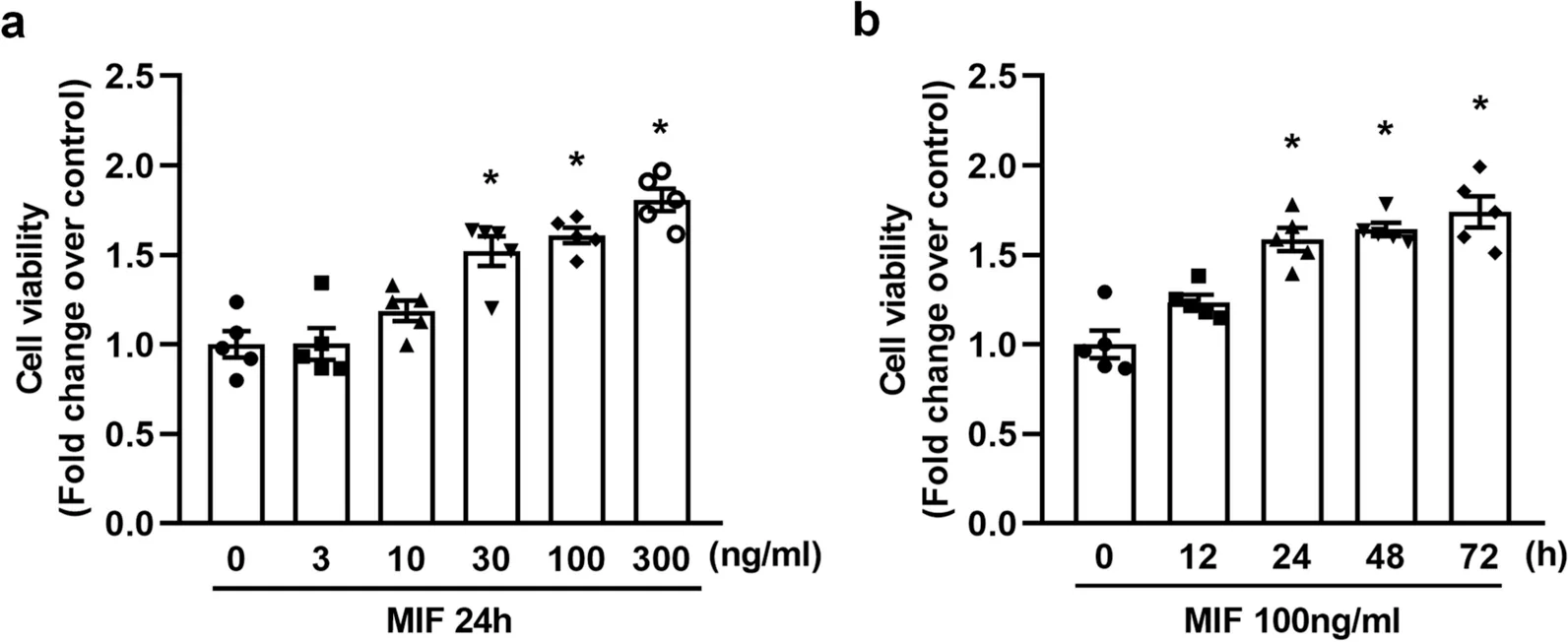
MIF promotes ASMCs proliferation. **a** ASMCs were exposed to different concentrations (0, 3, 10, 30, 100, 300 ng/ml) of MIF for 24 h, and cell viability was measured by Cell Counting Kit‐8 (CCK‐8) assay (n = 5 each group). **b** ASMCs were exposed to 100 ng/ml MIF for the indicated time (0, 12, 24, 48, 72 h), and cell viability was texted using CCK‐8 assay (n = 5 each group). *P < 0.05 vs. Control

To investigate the role of MIF inducing proliferation of ASMCs, cell viability was assessed by CCK-8 assay after treating ASMCs with different concentrations (0, 3, 10, 30, 100, 300 ng/ml) of MIF for 24 h. Figure 1a showed that cell viability was enhanced with increasing MIF concentration; 100 ng/ml of MIF triggered a 1.61-fold increase in viability of ASMCs at 24 h compared to the control (P < 0.05). Based on the result and previous studies [6], 100 ng/ml MIF was used in subsequent cell experiments. Figure 1b showed that MIF (100 ng/ml) promoted ASMCs proliferation in a time‐dependent manner.

### ERK1/2 mediates MIF-induced Drp1 phosphorylation, autophagy activation and E-cadherin downregulation in ASMCs

**Fig. 2:**
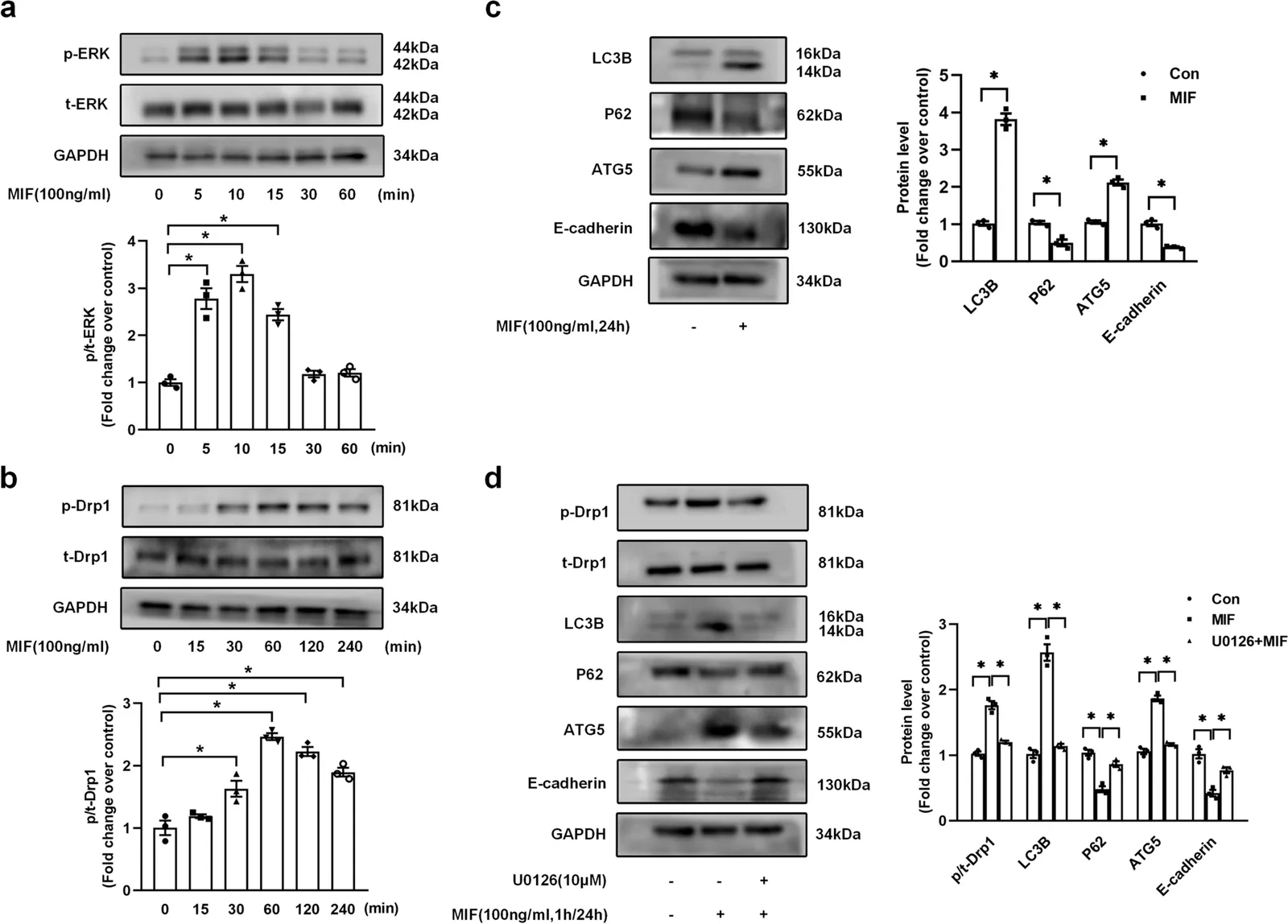
ERK1/2 mediates MIF-induced Drp1 phosphorylation, autophagy activation and E-cadherin downregulation in ASMCs. **a** MIF (100 ng/ml) remarkably increased ERK1/2 phosphorylation level time-dependently in ASMCs, and p‐ERK1/2 and t‐ERK1/2 levels were evaluated by immunoblotting (n = 3 each group). **b** MIF (100 ng/ml) time‐dependently elevated the phosphorylation level of Drp1 Ser616 in ASMCs, and p‐Drp1 and t‐Drp1 levels were evaluated by immunoblotting (n = 3 each group). **c** MIF (100 ng/ml, 24 h) activated autophagy and downregulated E-cadherin, and protein levels of LC3B, P62, ATG5 and E-cadherin were assessed by immunoblotting (n = 3 each group)**. d** ASMCs were pre‐treated with 10 μM U0126 for 30 min and then stimulated by 100 ng/ml MIF for 1 h or 24 h, and protein levels of p‐Drp1, t‐Drp1, LC3B, P62, ATG5 and E-cadherin were assessed by immunoblotting (n = 3 each group). For original blot images, see Additional file 1. *P < 0.05

Previous studies have shown that MIF promotes proliferation of a variety of cells by activating ERK1/2 signaling pathway [34, 35]. To explore the mechanisms underlying MIF-induced ASMC proliferation, we examined the phosphorylation level of ERK1/2. 100 ng/ml MIF was used to stimulate ASMCs for different times (0, 5, 10, 15, 30 and 60 min), and Fig. 2a showed that MIF remarkably increased ERK1/2 phosphorylation level time-dependently in ASMCs with the maximal effect at 10 min. Next, we determined the specific change of Drp1 in ASMCs after MIF stimulation. As shown in Fig. 2b, MIF time-dependently increased the phosphorylation level of Drp1 Ser616 in ASMCs with the most pronounced effect at 1 h. However, the total expression of Drp1 remained unchanged. To explore whether MIF can activate autophagy in ASMCs, we used immunoblotting to detect the expression of autophagy-related proteins. Figure 2c showed that MIF increased LC3B and ATG5 expressions, and reduced P62 expression, which indicated that autophagy was significantly activated. Furthermore, E-cadherin was also down-regulated after treatment of ASMCs with MIF. Collectively, these results indicate that MIF promotes phosphorylation of ERK1/2 and Drp1, autophagy activation and E-cadherin downregulation in ASMCs.

To further investigate whether ERK1/2 mediated MIF-induced Drp1 phosphorylation, autophagy activation and E-cadherin downregulation, we pre-intervened ASMCs with ERK1/2 inhibitor U0126 (10 μM) for half an hour before stimulation with MIF (100 ng/ml, 1 h or 24 h). Figure 2d showed that after suppression of ERK1/2, MIF-induced the increase of Drp1 phosphorylation was significantly reversed, and the changes of expression of LC3B, ATG5, P62 and E-cadherin were dramatically restored. These results suggest that ERK1/2 is involved in MIF‐induced Drp1 phosphorylation, autophagy activation and E-cadherin downregulation in ASMCs.

### Drp1 mediates MIF induction of E-cadherin downregulation by autophagy activation

**Fig. 3:**
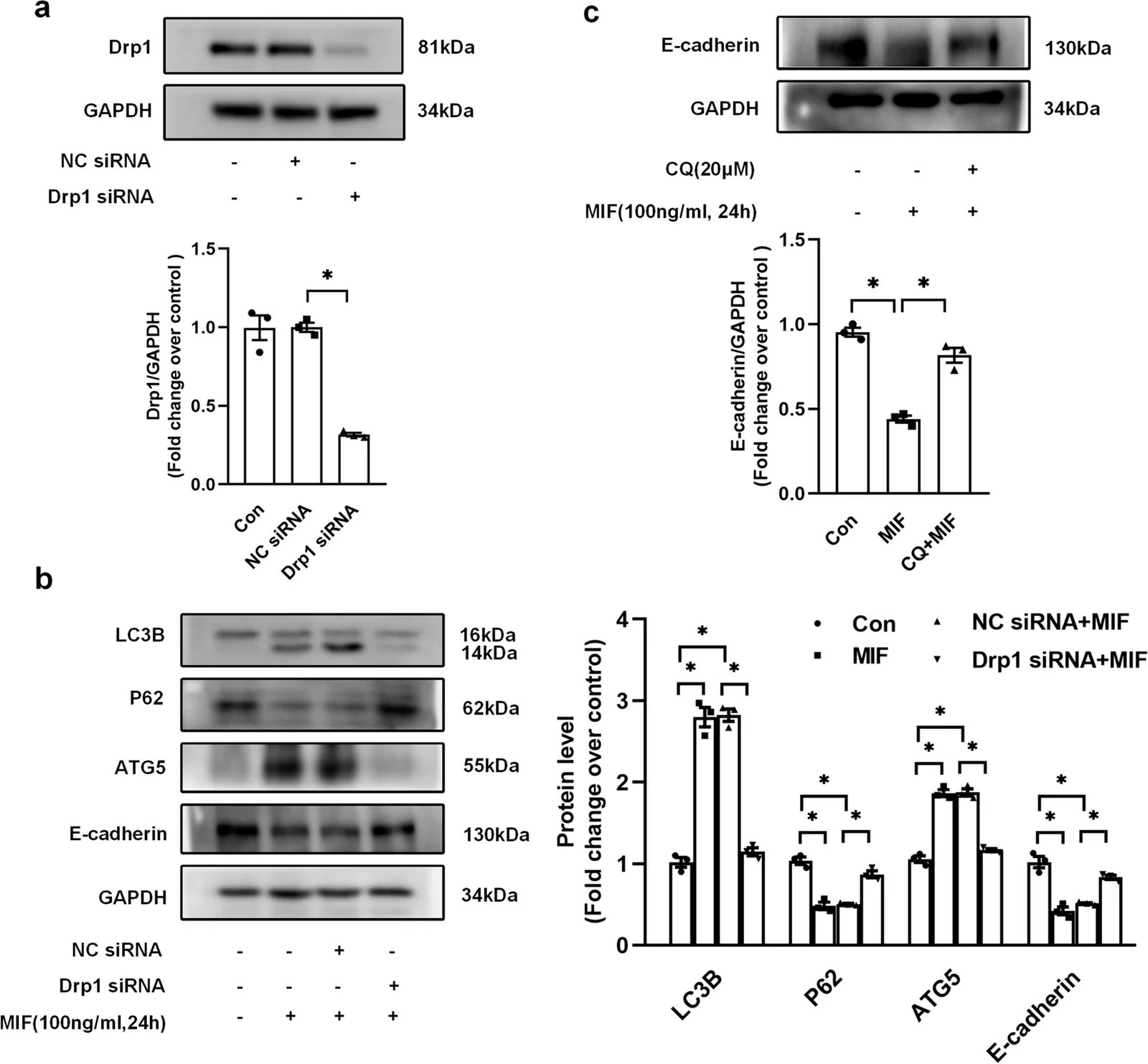
Drp1 mediates MIF induction of E-cadherin degradation by autophagy activation. **a** The silencing effect of Drp1 was evaluated by western blotting after transfection of Drp1 siRNA or NC siRNA for 48 h in ASMCs (n = 3 each group). **b** ASMCs were exposed to 100 ng/ml MIF for 24 h after the transfection of Drp1 siRNA for 24 h. Protein levels of LC3B, P62, ATG5 and E-cadherin were measured by western blotting (n = 3 each group). **c** ASMCs were pre‐treated with 20 μM CQ for 1 h and then stimulated with 100 ng/ml MIF for 24 h. Expression of E-cadherin protein was examined by immunoblotting (n = 3 each group). For original blot images, see Additional file 1. *P < 0.05

To investigate whether Drp1-driven autophagy activation mediates MIF-induced downregulation of E-cadherin in ASMCs, we pre-silenced Drp1 by siRNA or applied the autophagy inhibitor CQ. Figure 3a showed the Drp1 protein level was reduced to 32% of control after transfection with Drp1 siRNA in ASMCs for 48 h, whereas transfection with NC siRNA did not affect Drp1 protein level. As shown in Fig. 3b, pretreatment of cells with Drp1 siRNA reversed the MIF-induced activation of autophagy and the reduction of E-cadherin. Furthermore, MIF-induced E-cadherin downregulation was preserved after pre-intervening cells with autophagy inhibitor CQ (Fig. 3c). Overall, these results suggest that Drp1 mediates MIF-induced autophagy activation in ASMCs, which further leads to E-cadherin downregulation.

### ERK/Drp1-mediated autophagy activation is involved in MIF-induced proliferation of ASMCs

**Fig. 4:**
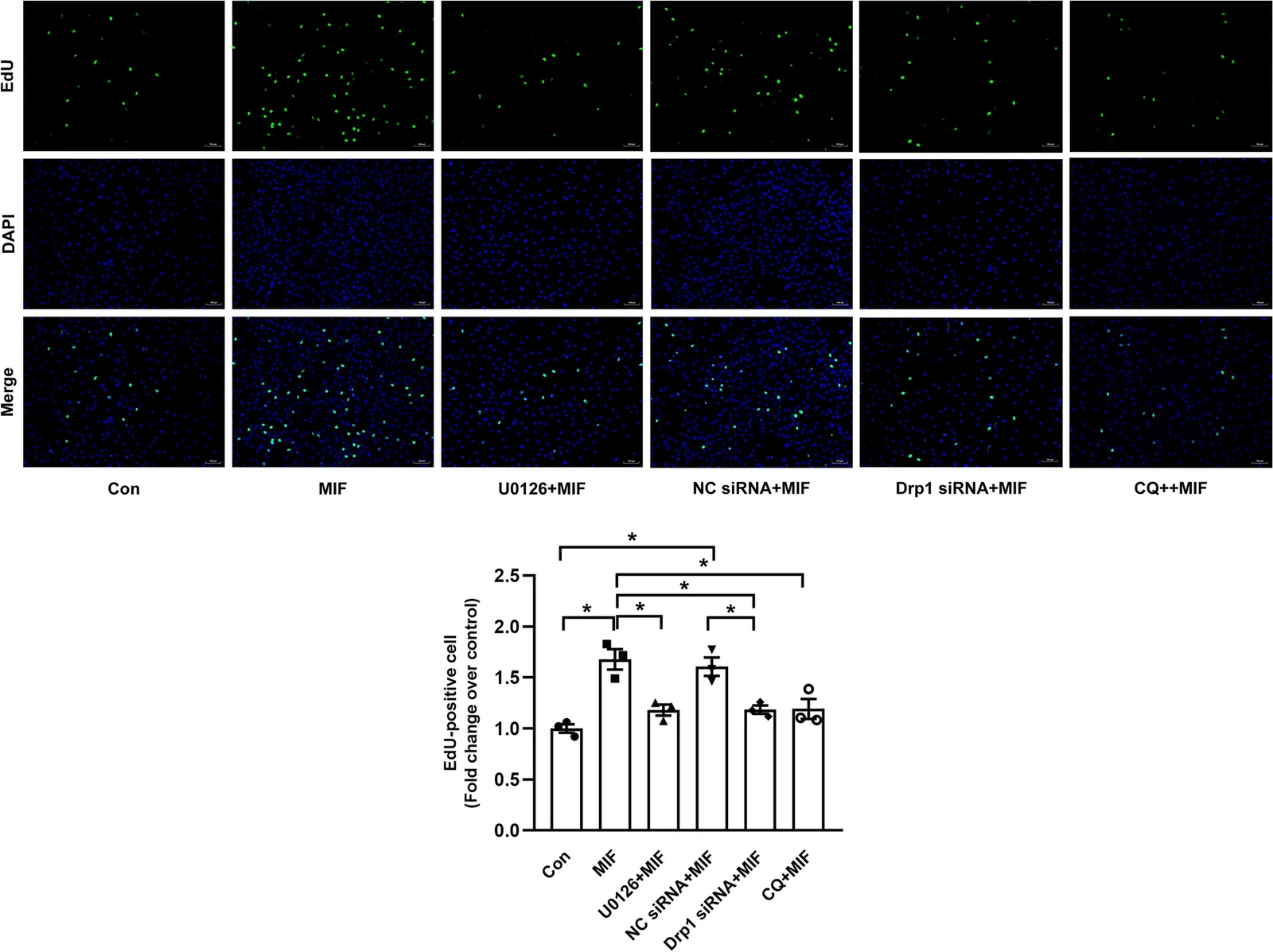
MIF stimulates ASMCs proliferation via ERK/Drp1 mediated autophagy activation. ASMCs were transfected with Drp1 siRNA or NC siRNA for 24 h, or pre‐treated with 10 μM U0126 for 30 min or 20 μM CQ for 1 h, and then incubated with 100 ng/ml MIF for 24 h. Cell proliferation was detected by EdU incorporation assay (scale bar = 100 μm, n = 3 each group). *P < 0.05

To clarify whether ERK/Drp1-mediated autophagy activation is associated with MIF-induced proliferation of ASMCs, we first priorly treated cells with ERK1/2 inhibitor U0126 (10 μM, 30 min), Drp1 siRNA (24 h) or autophagy inhibitor CQ (20 μM, 1 h), and then stimulated ASMCs with 100 ng/ml MIF for 24 h; cell proliferation was evaluated by 5′-ethyl-2′-deoxyuridine (EdU) admixture assay. As shown in Fig. 4, respective interference with ERK, Drp1 and autophagy significantly inhibited MIF-induced ASMCs proliferation as assessed by EdU staining, and the percentage of EdU-positive cells decreased from 1.67-fold to 1.18-fold, 1.19-fold, and 1.25-fold in MIF-treated cells, respectively. These results suggest that MIF promotes proliferation of ASMCs via ERK/Drp1 mediated autophagy activation.

### MIF inhibitor 4-IPP attenuates airway remodeling in OVA-induced asthma model by suppressing ERK1/2/Drp1 mediated autophagy activation

**Fig. 5:**
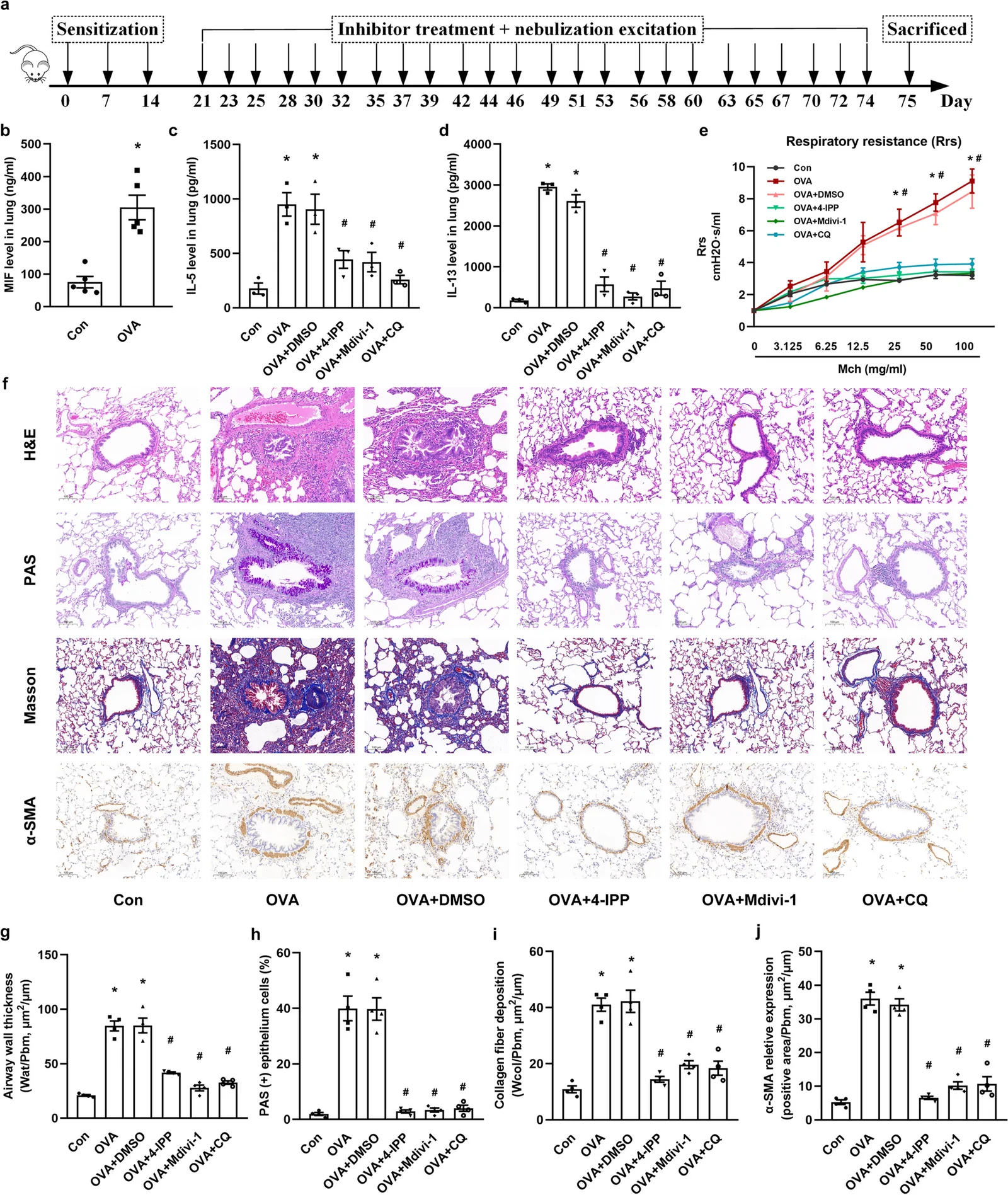
MIF inhibitor 4-IPP alleviates the development of OVA‐induced rats asthma model. **a** A schematic diagram of the OVA-sensitized chronic asthmatic rat model (n = 5 each group). **b** Concentrations of MIF in rat lung tissues were measured by Elisa (n = 5 each group). Concentrations of IL-5 **(c)** and IL-13 **(d)** in rat lung tissues were measured using Elisa (n = 3 each group). **e** Respiratory resistance (Rrs) was measured with inhalation of designated doses of nebulized methacholine chloride (0–100 mg/ml) using a FlexiVent animal ventilator (n = 3 each group). **f** Bronchial wall thickness shown by haematoxylin and eosin (H&E) staining, epithelial goblet cell proliferation shown by Periodic Acid-Schiff (PAS) staining, subepithelial collagen fiber deposition shown by Masson staining and bronchial muscularization shown by α‐smooth muscle actin (α‐SMA) staining, scale bar = 100 µm. **g** Changes in airway wall thickness (Wat/Pbm) (n = 4 each group). **h** Changes of PAS-positive epithelium cells (%) (n = 4 each group). **i** Changes of subepithelial collagen fiber deposition (Wcol/Pbm) (n = 4 each group). **j** Muscularization quantification (α-SMA positive area/Pbm) (n = 4 each group). *P < 0.05 vs. Control; ^**#**^P < 0.05 vs. OVA

On the basis of cellular experiments, we established an ovalbumin (OVA)-induced asthma rat model to verify whether the above in vitro mechanisms were also involved in asthma development. As shown in Fig. 5b, OVA-induced asthma model revealed significantly higher MIF concentration in the lung tissues than the control group. After treatment with the novel irreversible inhibitor and the specific suicide substrate of MIF, 4-iodo-6-phenylpyrimidine (4-IPP), the concentrations of Th2-type cytokines, IL-5 and IL-13, in lung tissues were significantly reduced (Fig. 5c and d). Meanwhile, respiratory resistance (Rrs) to methacholine increased dose-dependently in OVA-induced mice, but remarkably repressed after 4-IPP treatment (Fig. 5e). In addition, bronchial wall thickness, epithelial goblet cell proliferation, subepithelial collagen fiber deposition and bronchial muscularization were suppressed in 4-IPP-treated OVA-asthmatic rats (Fig. 5f–j). In summary, MIF inhibitor 4-IPP treatment prevented airway remodeling in the OVA-induced asthma rat model.

**Fig. 6:**
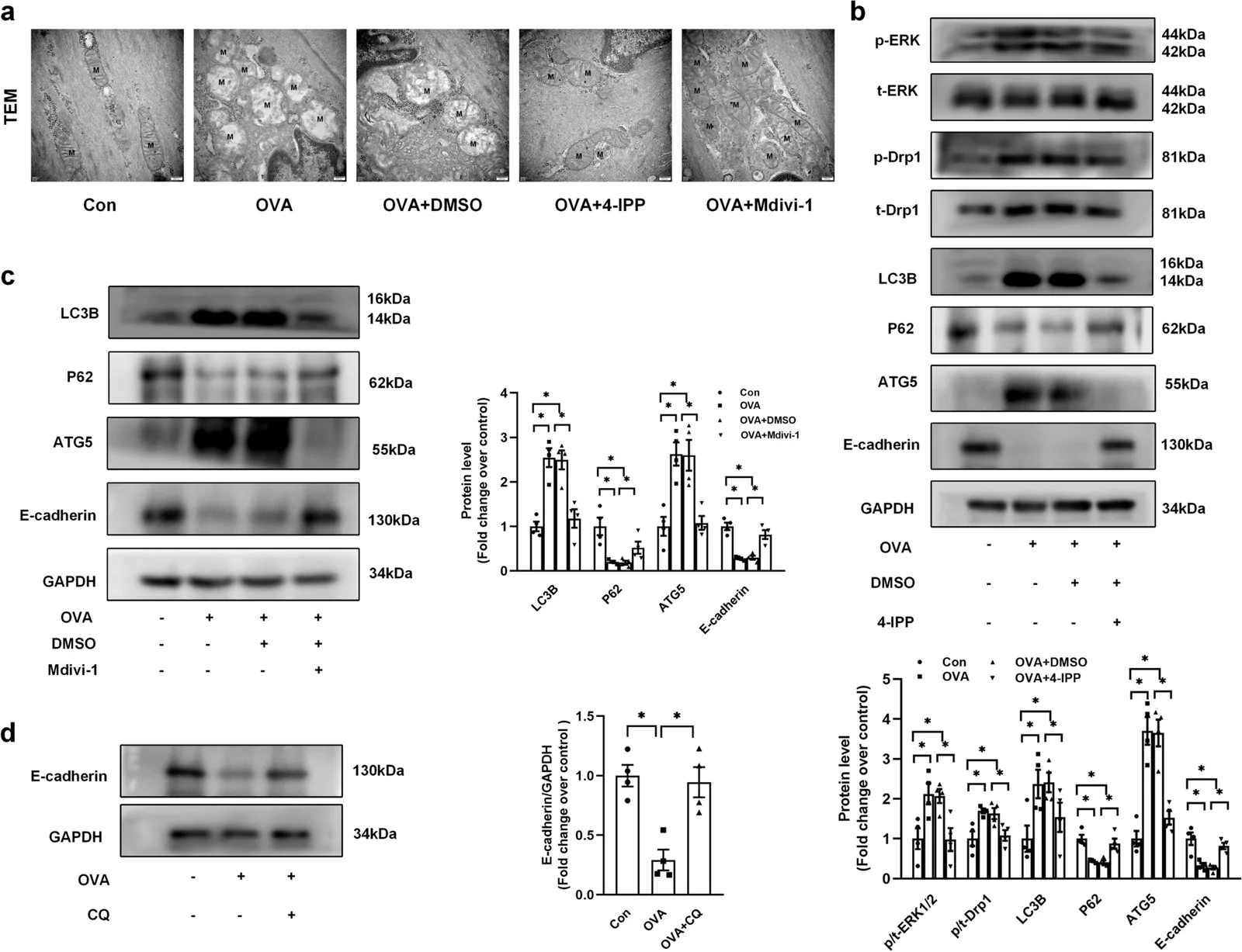
Relevant interventions prevent mitochondrial pathological changes and regulate their downstream molecules phosphorylation or expressions in OVA‐induced asthma rats. **a** Overview of the ultrastructure of mitochondria in ASMCs of OVA‐induced asthma rats by transmission electron microscopy (TEM), scale bar = 200 nm. M, mitochondria. **b** Proteins levels of p-ERK, t-ERK, p‐Drp1-Ser616, t‐Drp1, LC3B, P62, ATG5 and E-cadherin in lung tissues were assessed by immunoblotting (n = 4 each group). **c** Protein levels of LC3B, P62, ATG5 and E-cadherin in lung tissues (n = 4 each group). **d** Protein level of E-cadherin in lung tissues (n = 4 each group).  For original blot images, see Additional file 1. *P < 0.05

Next, we examined the phosphorylation levels of ERK1/2 and Drp1 in the lung tissues of OVA-induced asthmatic rats, and the results showed that the phosphorylation levels of ERK1/2 and Drp1 Ser616 were highly elevated. In addition, OVA-induced asthmatic rats presented obviously rounder and shorter mitochondria, apparently water-filled and swollen with disorganized or absent mitochondrial cristae structure in ASMCs compared with the control group. Meanwhile, autophagy activation, including increased expression of LC3B and ATG5 as well as decreased expression of P62, and down-regulated expression of E-cadherin were observed in OVA rats. However, after 4-IPP administration, the above changes were reversed in OVA-asthmatic rats (Fig. 6a and b).

### Inhibition of mitochondrial fission and autophagy suppresses airway remodeling and asthma development by reversing E-cadherin expression in rats

To explore whether Drp1-dependent mitochondrial fission and subsequent autophagy activation mediate airway remodeling and asthma development, we applied Mdivi-1 (a kind of mitochondrial division inhibitor) and the autophagy blocker CQ in OVA-induced asthmatic rats. Compared with the OVA group, Mdivi-1 or CQ effectively reduced IL-5 and IL-13 concentrations in lung tissues and attenuated airway resistance (Fig. 5c–e). In addition, increased bronchial wall thickness, epithelial goblet cell proliferation, subepithelial collagen fiber deposition and bronchial muscularization were alleviated in Mdivi-1-treated and CQ-treated asthmatic rats (Fig. 5f–j). Next, transmission electron microscopy examined the structural changes of mitochondria in ASMCs of OVA-induced asthmatic rats after Mdivi-1 treatment. The results showed that Mdivi-1 treatment inhibited OVA-induced mitochondrial alterations (Fig. 6a) and autophagy activation indicated by down-regulated expression of LC3B and ATG5, and up-regulated expression of P62. In addition, Mdivi-1 also increased E-cadherin expression in the OVA-asthma model (Fig. 6c). Similarly, administration of CQ treatment also reversed the downregulation of E-cadherin (Fig. 6d). To sum up, these findings suggest that inhibition of Drp1 suppresses autophagy activation, further blocking E-cadherin downregulation, thereby preventing OVA-induced asthma development and airway remodeling.

## Discussion

In the present study, we elucidated the role and mechanisms of MIF in promoting airway remodeling in asthma. We demonstrated that MIF increased Drp1 phosphorylation through the activation of the ERK1/2 signaling pathway, which subsequently stimulated autophagy activation and further led to the downregulation of E-cadherin, ultimately promoting the proliferation of ASMCs and airway remodeling in asthma.

Macrophage migration inhibitory factor (MIF) is an important pro-inflammatory cytokine, multifunctional immunomodulator and cytokine with diverse functions involved in a variety of pathologies, including inflammatory responses [36], angiogenesis [37], cell proliferation [38], autophagy [39], and glucocorticoid resistance [40]. Previous studies have demonstrated that MIF activates the Src-family protein kinases, mitogen-activated protein kinase (MAPK), PI3K-Akt cascade signaling pathway, NF-κB pathway, and inhibits p53 mainly by binding to the CD74/CD44 complex [41–44]. MIF is known to have direct effects on T cell activation and acts on ILC2 cells to release type 2 cytokines, including IL-5 and IL-13 [45, 46]. Our results indicated that MIF could promote the proliferation of ASMCs, phosphorylated ERK and Drp1, activated autophagy and downregulated E-cadherin in vitro. Meanwhile, MIF remarkably elevated in lung tissues of OVA-induced asthma rat model, accompanied with airway inflammation and excessive airway remodeling. The first small molecule MIF inhibitor to be described is ISO-1, which binds to the MIF tautomerase active site and inhibits downstream signaling [47] and has been shown to inhibit OVA and HDM-induced airway remodeling in a mouse model of asthma [48, 49]. As a new MIF-specific suicide substrate and irreversible inhibitor, the small molecule antagonist 4-IPP binds covalently to the N-terminal proline at the MIF reciprocal enzyme sites and inhibits signal transduction. Recently, 4-IPP has been shown to block MIF/receptor interactions and to be more effective than ISO-1 in preventing migration, and invasion in human cancer cell lines through MAPK and NF-κB signals [28, 50, 51]. Moreover, 4-IPP has shown good therapeutic effects in rheumatoid arthritis through its capacity of attenuation of the MAPK/COX2/PGE2 signaling cascade [11] and 4-IPP treatment significantly decreases the expression of TGF-β1 in joint capsule fibroblasts that attenuates joint capsule inflammatory cell infiltration [52, 53], which indicating the important role of 4-IPP in inflammation diseases. In the present study, the use of the MIF inhibitor 4-IPP significantly reduced Th2 inflammatory factors (IL-5 and IL-13) production, respiratory resistance as well as airway remodeling in OVA-induced asthmatic rat models via ERK/Drp1 pathway and autophagy activation, showing effective drug potential to alleviate airway hyperresponsiveness and asthma progression. However, the specific mechanisms of 4-IPP for repressing Th2-type inflammation and asthma treatment still need to be further explored.

Drp1 is a member of the GTPases kinetic protein family and is a key regulator of mitochondrial fission. When the phosphorylation level of Drp1 Ser616 site is elevated, Drp1 is activated and translocated from cytoplasm to outer mitochondrial membrane inducing mitochondrial division and fragmentation, thus suppressing cell death, which is a new marker of proliferative diseases [54]. Zhang et al. have demonstrated that lipopolysaccharide promotes ASMCs proliferation by enhancing Drp1 Ser616 phosphorylation level thereby triggering abnormal mitochondrial fission [12]. Several studies have linked Drp1-mediated mitochondrial fission to ERK1/2 activation, thus linking Drp1 activation to enhanced inflammatory responses and proliferation of multiple cell types in response to various stimuli [20, 55–57]. However, there is a lack of sufficient understanding of the role of Drp1 in airway remodeling and regulatory mechanisms. In the present study, we showed that MIF significantly increased the phosphorylation level of Drp1 Ser616 in ASMCs via ERK1/2 activation, and knockdown of Drp1 inhibited MIF-induced proliferation of ASMCs. Furthermore, in OVA-asthma models, MIF inhibitor 4-IPP prevented AHR and airway remodeling by inhibiting Drp1 activation and Drp1-dependent mitochondrial fission.

Autophagy is a highly regulated catabolic process that uses lysosomal degradation to remove damaged organelles, misfolded proteins and act as a cytoprotective agent [58, 59]. In addition, autophagy also plays a role in severe asthma, as elevated level of autophagy in granulocytes of peripheral blood and sputum are found in patients with severe asthma compared to non-severe asthma and healthy controls [60]. It has been found that autophagic activity of ASMCs is observed to be upregulated in OVA-induced mice asthma model [61] and TGF-β1-induced autophagy promotes collagen and fibronectin production in ASMCs, exacerbating airway remodeling [62, 63]. All above illustrate that activated autophagy in ASMCs is closely associated with airway remodeling. Li et al. have demonstrated that MIF-mediated autophagy activation in ASMCs through its’ receptor CD74 participates in airway remodeling [6], however, the exact regulatory mechanisms are not clear. In the present study, we found that MIF triggered autophagy activation in ASMCs via ERK1/2-mediated Drp1 phosphorylation. Chloroquine phosphate (CQ) has capacity of inhibiting lysosomal function, leading to extensive blockade of autophagy [64] and shows specific suppressive effects on T-cells and Th1/Th2 inflammation through JNK/AP-1 signaling [65]. In HDM-sensitized mice, CQ has been proved to repress the levels of IgE, IL-4/IL-13 and TGF-β in BALF, thereby restoring ASMCs phenotype via the ROS-AKT pathway [66]. Our in vivo experiments further confirmed that autophagy was activated in OVA-induced asthma models, and the application of autophagy inhibitor CQ significantly reversed Th2 cytokines release (IL-5/IL-13), airway resistance and remodeling. On the other hand, MIF inhibitor 4-IPP and Drp1 inhibitor Mdivi-1 treatment decreased autophagy activity in rat lung tissues, and significantly alleviated airway remodeling in the OVA-asthma model. These results demonstrated that MIF could activate autophagy via the ERK/Drp1 axis in airway.

E-cadherin is a calcium-dependent cell adhesion molecule that plays a key role in epithelial cell behavior, tissue formation, and tumor suppression [67]. A study of pulmonary hypertension shows that exogenous stimuli promote pulmonary artery smooth muscle cell proliferation by activating autophagic lysosomal degradation of E-cadherin [23]. In previous studies of asthma, airway epithelial linking E-cadherin molecule is considered the “gatekeeper” of the airway mucosa [21], and when E-cadherin expression is disrupted it enhances signaling between epithelial cells and underlying immune and structural cells, which may lead to allergic sensitization and airway remodeling, including goblet cell hyperplasia, and smooth muscle cells conversion to a proliferative phenotype [68–70]. It has also been reported in the literature that IL-17 and neutrophils induce airway smooth muscle proliferation by activating neutrophil elastase-related E-cadherin/β-catenin signaling [71], and in HDM-induced asthma models, MIF increases airway responsiveness by causing the disruption and delocalization of epithelial E-cadherin to increase airway responsiveness [49]. In the present study, we found that MIF significantly reduced E-cadherin protein level in ASMCs through Drp1-mediated autophagy, and pharmacological inhibition of autophagy restored the decreased E-cadherin protein level in OVA-induced asthma model of rats. Taken together, our study suggests that MIF induces autophagy activation through ERK1/2-mediated Drp1 activation and subsequent mitochondrial fission, which further decreases E-cadherin expression and promotes proliferation of ASMCs, thereby promoting airway hyperresponsiveness and airway remodeling.

## Conclusions

In conclusion, we demonstrated the critical role of MIF in asthma through in vitro and in vivo experiments and elucidated that MIF promoted the proliferation of ASMCs via the ERK1/2/Drp1 axis mediated autophagy activation and consequent E-cadherin reduction. In addition, the application of MIF-specific suicide substrate and irreversible inhibitor 4-IPP effectively alleviated airway hyperresponsiveness and airway remodeling in OVA-induced asthma model rats by repressing aberrant mitochondrial fission-mediated autophagy activation.

## Supplementary Information

**Additional file 1. **The original western blotting images for Figs. 2, 3, and 6.

## Data Availability

The data that support the findings of this study are available from the corresponding author upon reasonable request.

## Abbreviations

AHR: Airway hyper-responsiveness
ASMCs: Airway smooth muscle cells
MIF: Macrophage migration inhibitory factor
OVA: Ovalbumin
4-IPP: 4-Iodo-6-phenylpyrimidine
Drp1: GTPase dynamin-related protein 1
ERK: Extracellular signal-regulated kinase
DMEM: Dulbecco’s modified eagle medium
FBS: Fetal bovine serum
α-SMA: α-Smooth muscle actin
CQ: Chloroquine phosphate
CCK-8: Cell Counting Kit-8
EdU: 5-Ethynyl-2′ -deoxyuridine
siRNA: Small interfering RNA
NC: Negative control
ELISA: Enzyme-linked immunosorbent assay
H&E: Hematoxylin and eosin
PAS: Periodic acid-Schiff
Wat: Total bronchial wall area
Pbm: Bronchial basement membrane perimeter
Wcol: Airway collagen fiber area
TEM: Transmission electron microscope
