# Correction: Macrophage migration inhibitory factor exacerbates asthmatic airway remodeling via dynamin-related protein 1-mediated autophagy activation

**DOI:** 10.1186/s12931-026-03906-w

**Published:** 2026-09-30

**Authors:** Jin Liu, Yuqian Chen, Huan Chen, Yan Wang, Danyang Li, Qianqian Zhang, Limin Chai, Yuanjie Qiu, Jia Zhang, Nirui Shen, Qingting Wang, Jian Wang, Manxiang Li

**Affiliations:** Department of Respiratory and Critical Care Medicine, The First Affiliated Hospital of Xi’an Jiaotong University, No. 277, West Yanta Road, Xi’an, Shaanxi 710061 People’s Republic Of China

**Correction: Respir Res 24, 216 (2023)**

**https://doi.org/10.1186/s12931-023-02526-y**

In this article [1], the author has identified an error in Fig. 5 of the published article. Specifically, during the assembly of Fig. 5f, the incorrect image was mistakenly uploaded in the Masson staining (Row 3) for the Con group, causing the original representative image belonging to the Con group to be incorrectly placed in the OVA + 4-IPP group, resulting in errors in the representative Masson staining images of the Con group and the OVA + 4-IPP group. The incorrect and correct images are displayed below.

Incorrect Figure 5:

**Fig. 5:**
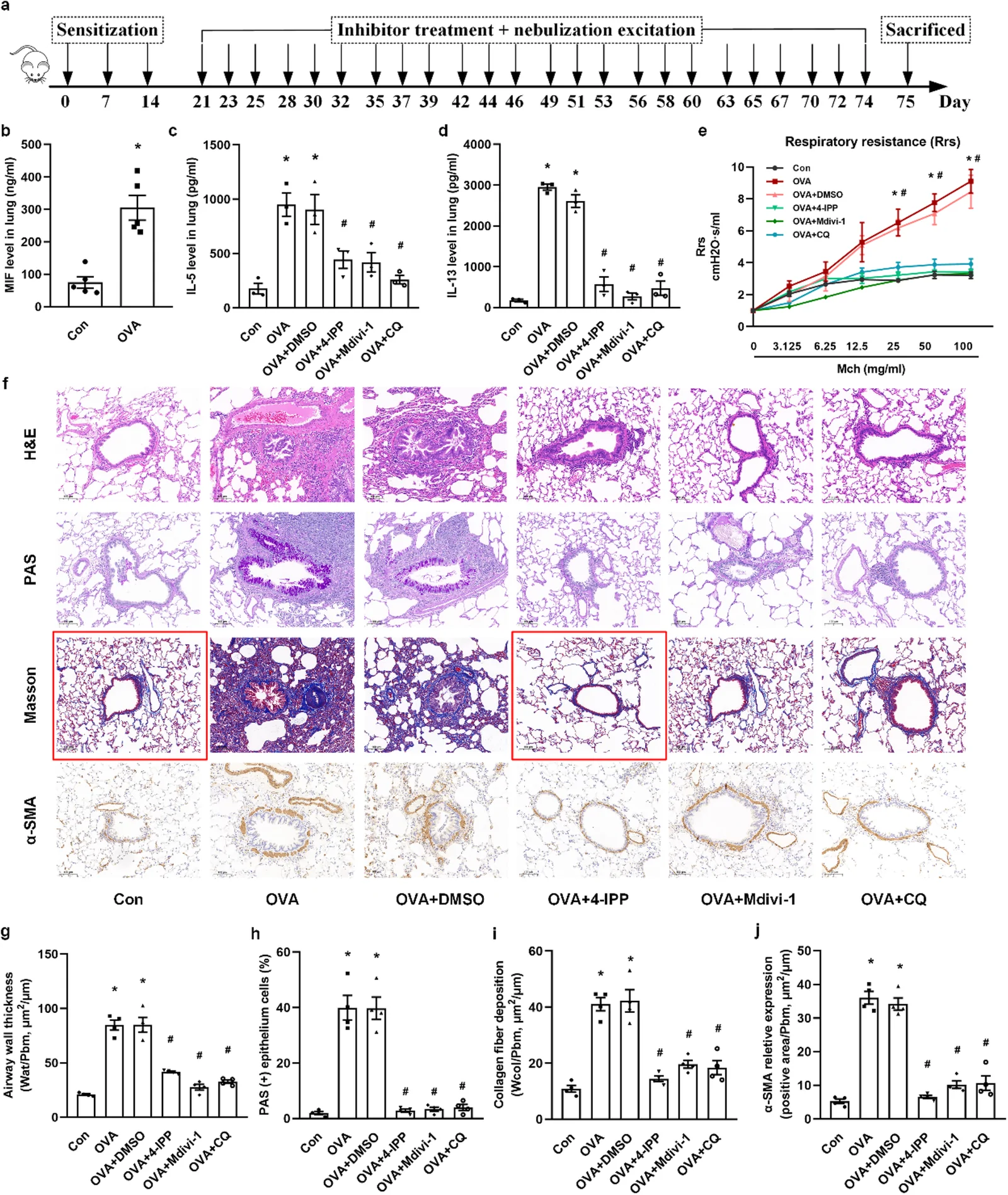
MIF inhibitor 4-IPP alleviates the development of OVA‐induced rats asthma model. **a** A schematic diagram of the OVA-sensitized chronic asthmatic rat model (*n* = 5 each group). **b** Concentrations of MIF in rat lung tissues were measured by Elisa (*n* = 5 each group). Concentrations of IL-5 (**c**) and IL-13 (**d**) in rat lung tissues were measured using Elisa (*n* = 3 each group). **e** Respiratory resistance (Rrs) was measured with inhalation of designated doses of nebulized methacholine chloride (0–100 mg/ml) using a FlexiVent animal ventilator (*n* = 3 each group). **f** Bronchial wall thickness shown by haematoxylin and eosin (H&E) staining, epithelial goblet cell proliferation shown by Periodic Acid-Schiff (PAS) staining, subepithelial collagen fiber deposition shown by Masson staining and bronchial muscularization shown by α‐smooth muscle actin (α‐SMA) staining, scale bar = 100 μm. **g** Changes in airway wall thickness (Wat/Pbm) (*n* = 4 each group). **h** Changes of PAS-positive epithelium cells (%) (*n* = 4each group). **i** Changes of subepithelial collagen fiber deposition (Wcol/Pbm) (*n* = 4 each group). **j** Muscularization quantification (α-SMA positive area/Pbm) (*n* = 4 each group). *P < 0.05 vs. Control; #*P* < 0.05 vs. OVA

Correct Fig. 5:

**Fig. 5:**
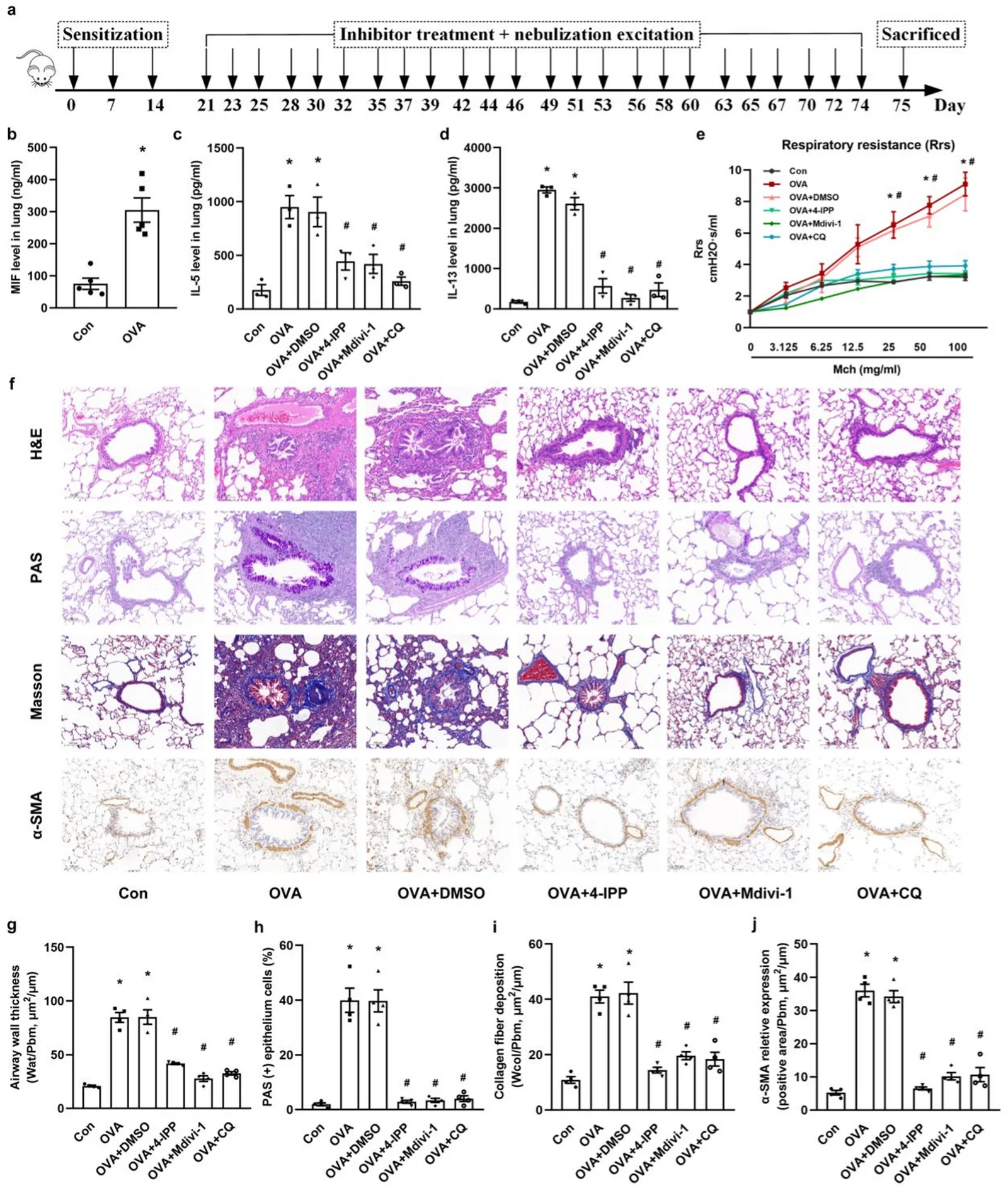
MIF inhibitor 4-IPP alleviates the development of OVA‐induced rats asthma model. **a** A schematic diagram of the OVA-sensitized chronic asthmatic rat model (*n* = 5 each group). **b** Concentrations of MIF in rat lung tissues were measured by Elisa (*n* = 5 each group). Concentrations of IL-5 (**c**) and IL-13 (**d**) in rat lung tissues were measured using Elisa (*n* = 3 each group). **e** Respiratory resistance (Rrs) was measured with inhalation of designated doses of nebulized methacholine chloride (0–100 mg/ml) using a FlexiVent animal ventilator (*n* = 3 each group). **f** Bronchial wall thickness shown by haematoxylin and eosin (H&E) staining, epithelial goblet cell proliferation shown by Periodic Acid-Schiff (PAS) staining, subepithelial collagen fiber deposition shown by Masson staining and bronchial muscularization shown by α‐smooth muscle actin (α‐SMA) staining, scale bar = 100 μm. **g** Changes in airway wall thickness (Wat/Pbm) (*n* = 4 each group). **h** Changes of PAS-positive epithelium cells (%) (*n* = 4each group). **i** Changes of subepithelial collagen fiber deposition (Wcol/Pbm) (*n* = 4 each group). **j** Muscularization quantification (α-SMA positive area/Pbm) (*n* = 4 each group). *P < 0.05 vs. Control; #*P* < 0.05 vs. OVA
