# Supplementary figures and images for: Macrophage migration inhibitory factor exacerbates asthmatic airway remodeling via dynamin-related protein 1-mediated autophagy activation
Source: Respir Res. 2023 Sep 6;24:216. doi: 10.1186/s12931-023-02526-y (PMC10481618; doi:10.1186/s12931-023-02526-y)

# Figure 2

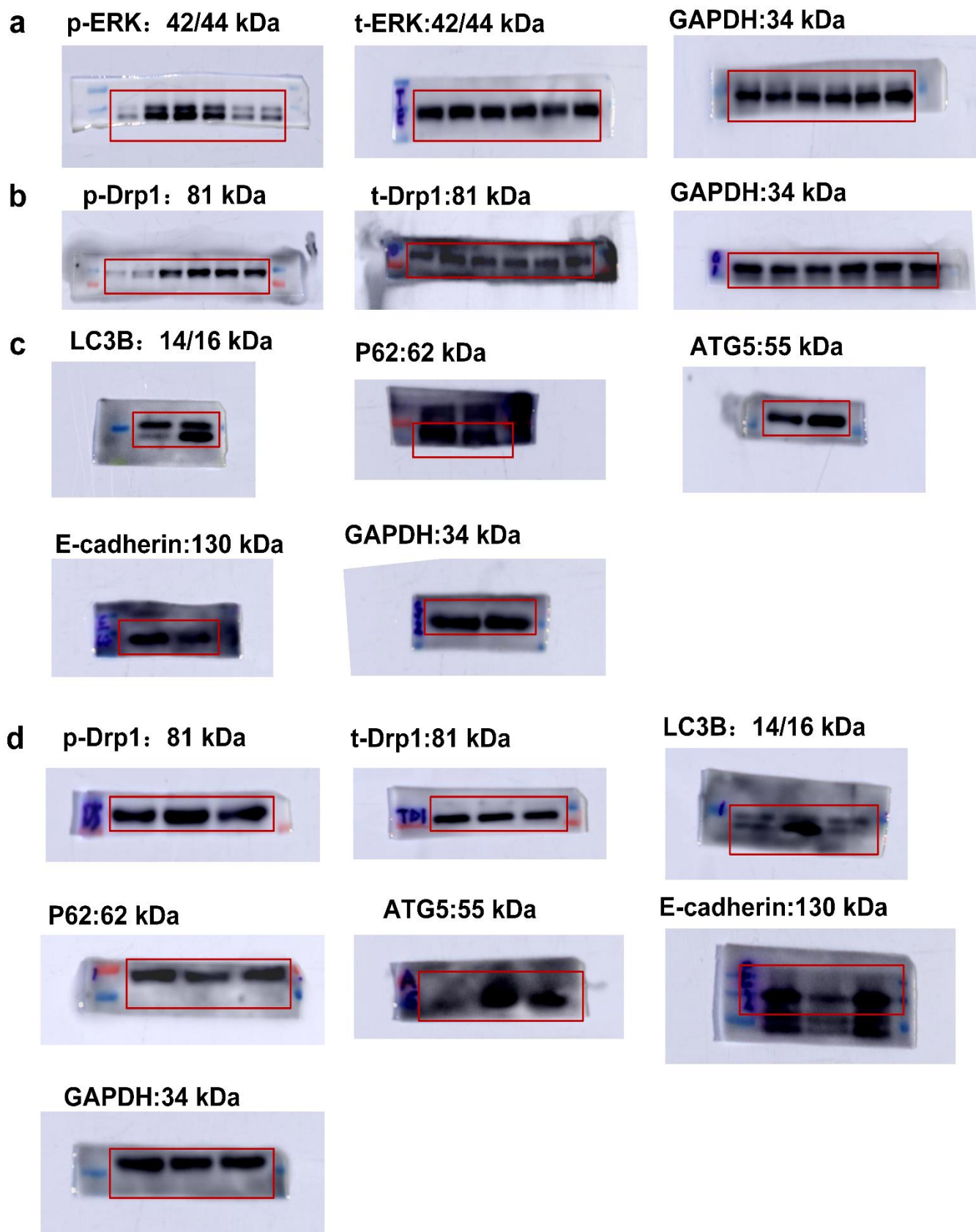

**Figure 3**

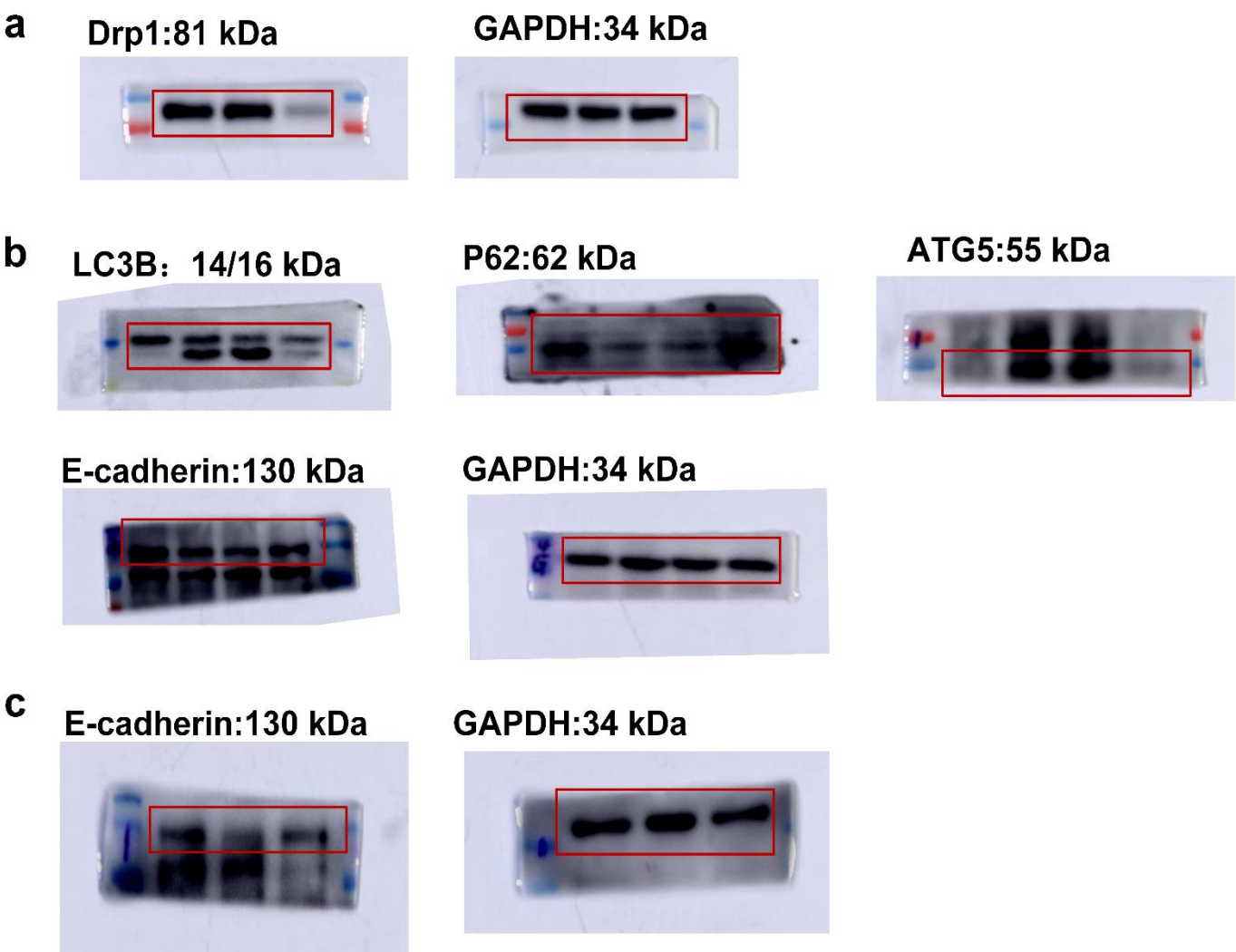

Figure 6

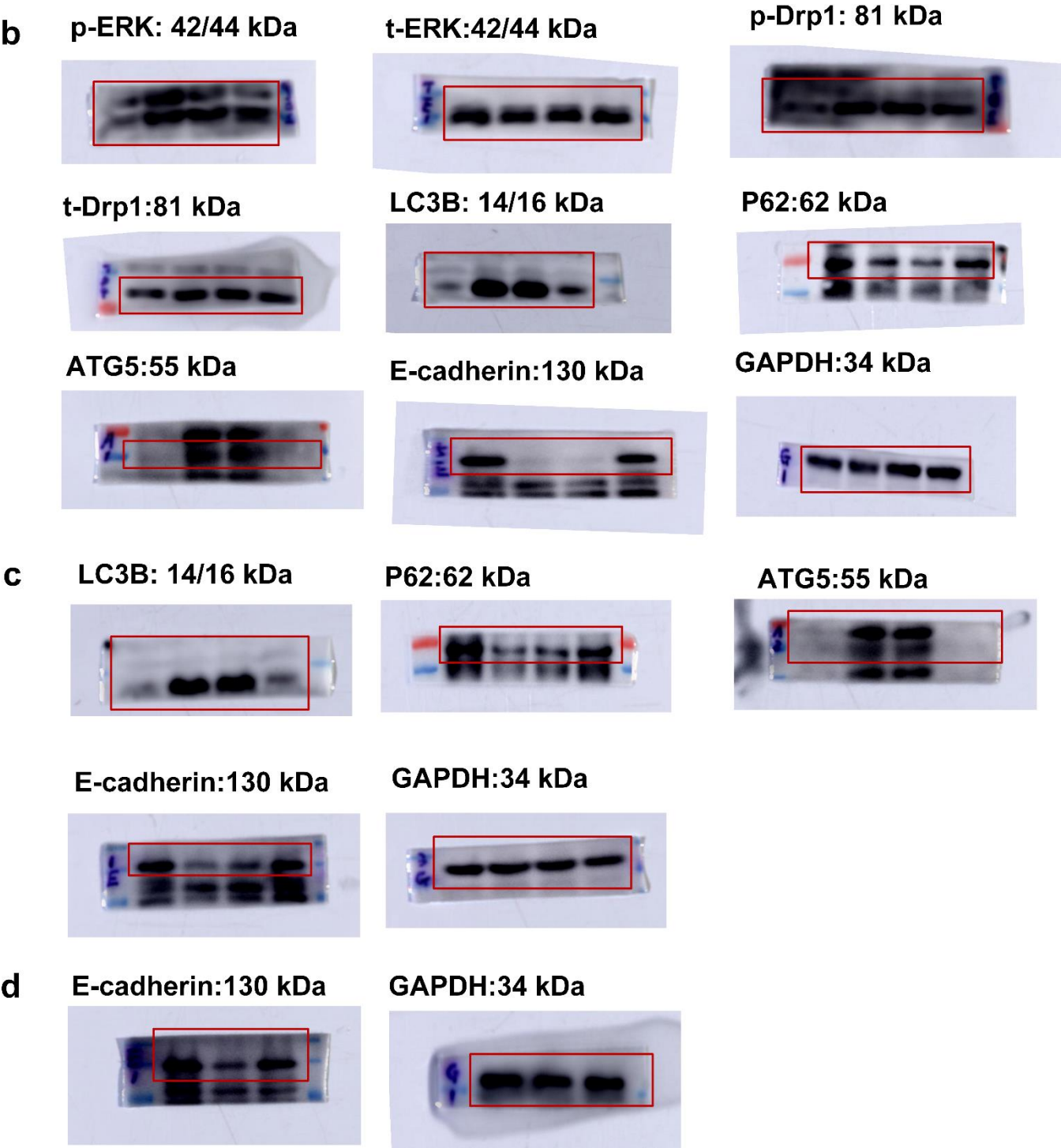

Supplement: Supplementary file 1 — Additional file 1. The original western blotting images for Figs. 2, 3, and 6. [file 12931_2023_2526_MOESM1_ESM.pdf]
